# 533. Blister Epidermal Grafting: Effect on Healing of Burn, Surgical and Chronic Wounds

**DOI:** 10.1093/jbcr/irag033.230

**Published:** 2026-04-06

**Authors:** Jeff Litt, Paul K Linneman

**Affiliations:** Burn and Reconstructive Center at Chippenham Hospital, Midlothian, VA; University of Missouri Health Care, Columbia, MO

## Abstract

**Introduction:**

Chronic wounds are a significant worldwide healthcare problem. Their direct costs are thought to exceed 25 billion dollars in the United States. Current standard wound-care treatments, while necessary for healing, are frequently not enough to attain timely wound closure. The current surgical technique of autografting is an operative procedure, requiring operating room time, anesthesia, and is associated with donor site pain and scarring. Epidermal grafting involves harvesting skin at the dermal-epidermal junction, and transplanting this onto a wound. Epidermal grafting has been reported to be beneficial in treatment of various patients including those with acute as well as chronic ischemic wounds.

A discontinued harvester for epidermal grafting using heat and suction to produce about 120 two mm diameter grafts covering 25 cm2 was developed, and utilized at a University Medical Center beginning in 2015. The grafting procedure can be performed in the clinic with no sedation, takes less than one hour, and the donor site is healed in one week with no scar. It was used for a variety of types of acute and chronic wounds until 2023 when it was removed from the market by the manufacturer. This review looks at the types of wounds treated, outcomes, and comparison with standard-of-care wound care treatment.

**Methods:**

A clinic registry was used to identify patients treated with the epidermal grafting procedure. Date of grafting, wound type, wound dimensions at time of graft as well as one month prior and at 1 & 2 months post-grafting were identified from the EMR. Wound closure was calculated as the percent reduction in wound area (length x width) at 1 & 2 months post-grafting compared with baseline at the time of graft. To compare with standard care, percent reduction from one month prior to grafting was calculated.

**Results:**

Between November 2015 and May 2023, 95 epidermal grafting procedures were identified. A variety of wounds were treated, predominantly burns & abrasions, surgical wounds and venous leg ulcers. All body parts were treated, with about half in the lower extremity. Wound size ranged from 1.5 to 126 cm2 (median 16.0). Standard of care treatment, reflected in % wound reduction during one month prior to graft, was 30%. Median reduction in wound size at one month post epidermal graft was 50% (*p*=.0006), and at 2 months was 82%.

**Conclusions:**

Use of the epidermal graft on a variety of acute and chronic wounds in aggregate was associated with a greater rate of wound closure compared with standard of care.

**Applicability of Research to Practice:**

With new technologies and regulatory changes influencing the practice and clinical decision-making in wound care practices, a reliable, effective, inexpensive, and easily-performed grafting techniques can be beneficial and efficacious. Reinstituting automated blister grafting could impact wound healing practices in the outpatient setting and improve results in the wound care population.

**Funding for the study:**

N/A.

